# Making sense of activity-based workplaces: perspectives from staff in a municipal setting

**DOI:** 10.1080/17482631.2026.2631081

**Published:** 2026-02-13

**Authors:** Erika Wall, Pär Löfstrand, Stig Vinberg, John Selander

**Affiliations:** a Department of Health Sciences, Mid Sweden University, Östersund, Sweden; b Department of Education, Psychology and Social work, Mid Sweden University, Östersund, Sweden

**Keywords:** Occupational wellbeing, organisational change, psychosocial work environment, sense-making, working conditions

## Abstract

**Purpose:**

This study explores how leaders and employees make sense of working conditions in a recently introduced activity-based workplace (ABW) in a municipal setting in Sweden. The analysis draws on Weick (1995) concept of sensemaking.

**Methods:**

Transcripts from focus group interviews were analysed using qualitative content analysis to examine how different staff groups experienced the transition from traditional cellular offices to ABW, and how these experiences framed their sense-making of ABW within the organisational context.

**Results:**

The analysis shows that ABW create working conditions marked by both increased interaction and persistent challenges related to privacy, confidentiality, noise, leadership, and ergonomics. Sensemaking was structured around five themes: Contrasting; Rationality; Collective process; Working from home; and Focusing on the ideal.

**Conclusion:**

Sensemaking of ABW is closely tied to how well concrete working conditions support core work tasks. Acceptance of ABW depends less on the concept itself and more on its ability to accommodate sector-specific demands, particularly confidentiality and focused work. The findings underscore the importance of aligning ABW design and implementation with everyday work practices in the public sector.

## Introduction

In the present study, we explore how experiences of working conditions after transitioning from traditional individual cellular offices to activity-based workplaces (ABWs) are made sense of in a municipal setting. Over the past decade, there has been a notable change in work environments and workplace cultures, with new perspectives on health and wellbeing at work in relation both time as space. In parallel with an overreaching digitalisation, a significant change has been the transition from traditional cellular offices to ABWs, a shift that has not only redefined the physical design of workplaces but has also created a new perspective on how work is organised and carried out.

In the literature, ABWs are defined and described somewhat differently across studies. Bäcklander et al. (2021) explain ABW as a flexibility concept centred on the office’s layout, offering a variety of spaces for work, such as smaller rooms for concentrated work, quiet zones, open lounge areas, and meeting rooms, while Bouvier and Eriksson (2014) recognise that descriptions of ABWs often include terms like quiet areas, open landscapes, formal and informal meeting places, workstations for projects, smaller rooms, and coffee areas. Despite these somewhat differing definitions of ABW, they seem to share a common mindset, i.e., that work is something that gets done, not a place people go to.

In the present study, we focus on how the employees in an organisation make sense of the change from cellular offices to an ABW. Rather than assessing ABW in terms of strengths and limitations in working conditions, the study examines the underlying processes through which meanings are constructed, negotiated, and stabilised in relation to this organisational change.

Previous research shows several different benefits when going from traditional individual offices to ABW environments. One central factor is the increased need for flexibility and collaboration in modern working life. Research by Truex et al. (2019) suggests that ABW environments promote creativity through increased information exchange. Studies also show that ABW environments encourage spontaneous collaboration and knowledge sharing among different teams and departments, fostering innovation and synergy (de Been et al., 2015). By creating an environment that encourages collaboration and openness, ABW environments might foster a more dynamic, engaging, and problem-solving workplace culture (Hua et al., 2020). A study by Richardson and West (2016) found that ABW layouts promote interdisciplinary interactions and knowledge sharing, fostering a culture of creativity. Similar results were found in a study by Stöllman et al. (2015).

Research also shows that ABW environments, to a greater extent than traditional offices, satisfy employees' needs and preferences, as well as their autonomy, which can stimulate creativity by allowing individuals to use the various work environments and manage their time in ways that enhance their creative process (Heerwagen & Kinzie, 2007). ABW environments can also contribute to more efficient resource utilisation (Jung et al., 2020). By implementing shared collaborative areas and flexible workstations, organisations can reduce office space and the overall amount of square footage needed per employee (Maina et al., 2019).

An additional factor that has driven the shift to ABW environments is the increased focus on employee health and well-being. Research shows that the physical work environment has a significant impact on employee health and productivity (Bodin Danielsson et al., 2015; Candido et al., 2019; Colenberg et al., 2020), and by offering a diversity of choices and work areas, ABW environments can create a more stimulating work environment that promotes employee well-being, satisfaction, and motivation (Bosch-Sijtsema et al., 2014; Nieuwenhuijsen & Topcu, 2019). ABW environments have the potential to facilitate social interaction and collaboration among employees, which can enhance social well-being and job satisfaction. Since ABW environments often feature ergonomic furniture, adjustable workstations, and natural lighting, and encourage movement throughout the workday by providing a variety of work settings and amenities such as standing desks, walking paths, and stairs, ABW has the potential to improve physical comfort and reduce the risk of musculoskeletal issues. Research by Hedge (2018) suggests that well-designed work environments, including ABW settings, can positively impact physical comfort and overall well-being. Such environments can reduce stress and burnout, which in turn can lead to reduced sick leave among employees (Nieuwenhuijsen & Topcu, 2019).

Despite the benefits of ABW environments, there are also challenges and difficulties that need to be addressed (Laing, 2017). One significant challenge is the need to balance the diverse needs and preferences of employees, such as the need for concentration. With numerous activities and interactions, employees may find it challenging to stay focused and avoid distractions, leading to decreased quality and work efficiency. Regarding the physical work environment in ABW, the different workstations, with their adjustable chairs and tables, might improve physical comfort, but the opposite could also be true. Studies show that employees experience adjusting office furniture (e.g., chairs and tables) as problematic (Pettersson-Strömbäck et al., 2018).

A further issue is that ABWs might compromise employees' privacy, making it difficult to concentrate on tasks requiring intense focus or hold confidential conversations (Laing, 2017; Seddigh et al., 2015). If there is insufficient variety or availability of workspaces, employees may struggle to find suitable spots to work, causing frustration and hindering productivity. Further, a study by Bodin Danielsson and Bodin (2008) found that employees in ABW environments experienced higher levels of stress due to noise and lack of privacy. In a recent systematic review by Masoudinejad and Veitch (2023) on the effects of ABWs on organisational productivity, the results were negative. Comparisons of ABWs to cellular offices favoured the cellular offices for all investigated categories, concluding that employees want and need spaces that support attention and focus. Organisations looking to save on property costs should weigh these savings against the overall effect of their design choices on organisational productivity. In summary, research shows that ABW environments is not only intended to create a more modern and attractive work environment but also to promote collaboration, creativity, well-being, and productivity among employees. However, when it comes to efficacy, work environment, and well-being, the research remains inconclusive.

Hence, a major challenge with regards to ABW environments has to do with their implementation. According to some literature (Eaton, 2019; Nieuwenhuijsen & Topcu, 2019), successful implementation of ABW involves careful planning, well-thought-out design, and professional management. If these aspects are lacking, the introduction of an ABW environment may face resistance from employees accustomed to traditional work setups (Maina et al., 2019). The implementation of ABW is also highlighted by Marzban et al. (2023). Their literature overview, which aimed to map and describe findings from research on ABW for the period 2010−2020, showed that the shortcomings of ABW environments were more related to how ABW is implemented and how occupants use it, rather than the concept itself. In the study it is hypothesised that many of the currently reported negative aspects of the ABW concept might diminish over time as ABW evolves and as new challenges arise. When it comes to implementing ABW, Babapour (2019) found that providing employees with continuous support and the opportunity to modify the workspace increased their appreciation of ABW. Brunia and De Been (2016) came to a similar conclusion, showing that employee satisfaction in ABWs was influenced by the implementation process. These studies highlight the importance of tailoring the ABW solution to the organisation’s culture, and when employees are invited to actively contribute to organisational decisions, the likelihood of success increases.

As this summarising overview of previous research demonstrates, most studies have focused on how activity-based working is perceived in terms of strengths and weaknesses. To our knowledge, no study has instead examined how employees make sense of the transition to ABW.

Against this background, the **aim** of the present study is to analyse how leaders and personnel in a municipal setting make sense-making of a newly introduced ABW as reflected in working conditions.

## Theoretical framework

Theoretically, we draw on Weick (1995) concept of sense-making as the basis for the analysis in the present study. Sense-making, in this specific case, refers to a process where individuals seek to clarify and understand what is going on when they encounter unexpected or confusing situations within the organisation (Weick, 1995), such as those related to disruptive times and spaces. Sense-making is a profoundly social process wherein individuals engage in the arguing and negotiation of meaning, striving to comprehend the organisational landscape and undertake collective actions (Berger & Luckmann, 1967; Maitlis, 2005; Weick et al., 2005). The social characteristics of the concept can be found in the seven key properties that are most often used when defining sense-making in organisations. Defined by Weick (1969, 1995), sense-making involves the construction of identity, retrospective analysis, enactment of meaningful environments, social interaction, continuous evolution, concentration on extracted cues, and, ultimately, it prioritises plausibility over precision. As described by Kihlberg (2022, p. 7–8), these properties manifest in organisations through three interconnected subprocesses. Initially, individuals discern and extract cues from their experiences, creating an initial understanding of the situation. Subsequently, they interpret this initial understanding, refining it into a more coherent and structured perception. Finally, they put these interpretations into practice, generating new iterations of the cycle (cf. Sandberg & Tsoukas, 2015, p. 19). These processes are key when distinguishing sense-making from other perspectives on how situations are understood. Hence, the process of sense-making implies that the individuals themselves are included in a relational process of creating the understanding of the situation (cf. Weick et al., 2005).

A major trigger for sense-making in organisations is thus intentional organisational change. Through structural reorganisations, present understandings of the organisation are challenged, generating confusion, uncertainty, and feelings of disorientation among the individuals within the organisation (Maitlis & Christianson, 2014; Maitlis & Sonenshein, 2010). Here, sense-making is understood as the consequence of changes in structures, responsibilities, and/or roles that give rise to uncertainty about the conditions for work (Lüscher & Lewis, 2008). Hence, organisational change most often entails uncertainties about the future (Balogun & Johnson, 2004, 2005; Maitlis & Lawrence, 2007). When encountering ambiguous, unexpected, or confusing situations, a process of sense-making arises where employees, in interaction with each other, develop an understanding of the previously unknown situation that has emerged (Maitlis & Christianson, 2014; Weick, 1995). However, as described by Sandberg & Tsoukas, (2015), the interactional part of the process must be understood in terms of intra-action. Thus, individual understanding cannot be transferred from one person to another but should be understood as a process in which various individuals intertwine their sense-making through discussions during everyday work. Hence, as exemplified by Kihlberg (Kihlberg, 2022, p. 8), how individuals react to the situation at hand provides information to others, who then react to it. In other words, through their reactions, people simultaneously influence each other's sense-making.

As described, the process of sense-making must be understood from a relational perspective. Hence, within interaction, individuals use their (mostly spoken) language to construct, share, and discuss how to understand the organisational change at hand (Hardy et al., 2005; Thomas et al., 2011; Tsoukas, 2005). Additionally, the experiences, beliefs, and pre-understandings held by the individual play a central part in this process. Thus, such pre-understandings form the basis for the process of sense-making (Balogun, 2006; Maitlis & Lawrence, 2007; Weick, 1995). In other words, from a constructive perspective, we change the context we live in as we speak; language constructs and changes reality (Potter & Wetherell, 1987).

However, the process of sense-making is complex and includes a wide range of perspectives that are discussed and negotiated during ordinary work. Hence, the process of sense-making does not take place in a vacuum but involves tensions and political dimensions of power (Weick, 1995) and can be described as a process where individuals at various levels within the organisation discuss, debate, and try to understand what is happening (Lawrence et al., 2012; Mumby, 2005; Thomas et al., 2011; Weick, 1995). Furthermore, the process of sense-making is related to other features of the context, including language, emotions, power, technology, leadership, and so on (Kihlberg, 2022; cf. Gephart, 1993; Maitlis & Christianson, 2014; Maitlis, 2005; Sandberg & Tsoukas, 2015; Smircich & Morgan, 1982; Weick, 1995), implying that social structures (i.e. formal and informal power, cf. Kihlberg, 2022; Maitlis & Christianson, 2014; Schildt et al., 2019) influence which perspectives become central in the process and which perspectives disappear (cf. Gephart, 1993). Also, in organisational change, various groups within the organisation might differ in terms of previous experiences, expectations, and so on. For this reason, groups might also differ in their understanding of the situation and take different sense-making positions, resulting in disparate and sometimes even opposite understandings between, for example, managers and employees (cf. Brown et al., 2008).

In previous research, it has been pinpointed that most studies on sense-making in organisations focus on the outcome of the collective understanding of organisational change; this obscures the variation of perspectives included in the process of sense-making, where different views are discussed and debated (Brown et al., 2015; Maitlis & Christianson, 2014; Sandberg & Tsoukas, 2015; Weick et al., 2005). It has been argued that the relevance of the different interests of various groups is being neglected (Schildt et al., 2019), and there has been a call for studies that include the influence of various actors in the analysis of sense-making in order to ‘clarify why major change initiatives unfold in a certain way’ (Kihlberg, 2022, p. 5). The present study contributes to filling this gap within the research on sense-making in organisations. More specifically, the study advances sense-making theory by showing how meaning is constructed and negotiated through concrete working conditions in everyday organisational practice. Using the theoretical perspective on sense-making, it is possible to demonstrate how interpretations of organisational change are anchored in material, spatial, and task-related conditions, such as confidentiality requirements, opportunities for focused work, and leadership presence. By including different groups of personnel within the same organisation, it is possible to capture both shared understandings and, eventually, divergent sense-making processes related to the recently completed transition from individual traditional cellular offices to ABW.

## Method

From the perspective of sense-making in organisations as a process where understanding is individually and collectively constructed through the use of language (Hardy et al., 2005; Thomas et al., 2011), a method was required that would consider not only individual views on the organisational change, but also how participants in dialogue discuss their meanings regarding working conditions in the new ABW setting. Consequently, a focus group interview study was conducted to enable an in-depth understanding of how different groups within the municipality comprehend the situation. We have previously used focus group interviews for data collection for the analysis of sense-making (Wall & Olofsson, 2008; Wall, 2014), which was found to be a sufficient method when interested in how the participants discuss the specific situation at hand.

The present study is part of a larger collaborative project named ‘Activity-based offices within the municipality of Östersund—a research and evaluation study’ (grants No MIUN 2021/1080, MIUN 2023/1753), which involves researchers in Psychology and Occupational Health Science together with a specific organisation, a mid-sized municipality in Sweden. At the end of 2023, the employees of the municipality moved into a newly constructed office featuring an ABW. As only 60% of the workforce had desk space, remote work was required. The move aimed to establish ABW practices, build support among employees, and align the building’s functionality with the ABW model. Additionally, the objective was to create an appealing and efficient workplace through advanced facilities and IT solutions, and at the same time save space and, thereby, money. A total of 620 staff members, including 534 employees and 86 managers, relocated to the building, representing various departments such as social care, administration, urban planning, and culture. The workforce was distributed across three floors, equipped with open-plan offices, creative, middle, and silent zones, conference rooms, a canteen, and coffee areas. The relocation process began in 2019 and included workshops for staff, managerial visits to other organisations using ABWs, and training in open-office environments, and was completed in 2024.

### Data collection, participants, and material

During the period of February (approx. two months after the relocation to ABW)—October 2024, five focus group interviews were conducted. One interview with individuals (*n* = 5) who held different leadership positions (HR, IT, economy, local strategy, and administration) in the municipality during the transition to the ABW, one interview with managers (*n* = 5) at different municipal departments (including HR, the municipality office, the cultural department, the business development office, and the community development administration), two interviews with employees (*n* = 4 + 5) from these different departments, and finally, one interview with employees solely from the social service (*n* = 6). The reason for interviewing representatives from different hierarchical levels separately, was to avoid risk biased communication. Participants were recruited by an assigned contact person within the municipality. Among the 25 individuals who participated in the five focus groups, 22 were women and 3 were men which reflects the gender distribution among employees.

An interview guide was used for conducting the interviews. This procedure involved thematic questions designed to create conversation regarding the research themes based on the participants’ experiences and/or thoughts about these themes, rather than on their assessment of their current work situation and working conditions. The interview guide was developed specifically for this study. The guide was organised into three overarching thematic sections: Background and motives for the transition to ABW; Implementation and current experiences of the ABW, and Future perspectives and anticipated challenges. Within each section, open-ended questions were used to stimulate collective reflection with focus on working conditions in the newly introduced ABW, but also on decision rationales, underlying assumptions, perceived benefits and risks, perspectives on leadership and communication, and the integration with broader organisational processes.

The interviews were conducted by two of the researchers in the team (first and last author), both with experience in qualitative and organisational research. Each focus group interview followed the same structure. At the outset, the moderators introduced the study aim, clarified that the interview was exploratory rather than evaluative, and emphasised that the interest lay in shared reasoning and groups reflections rather than individual positions. Participants were also informed about audio recording and confidentiality, and consent was obtained.

During the interview session, that lasted an average of 90 minutes, the moderators encouraged interaction among participants to elicit collective sense-making and different viewpoints within the group. One of the researchers (last author) took primary responsibility for facilitation, while the other (first author) focused on follow-up questions and monitoring group dynamics. To ensure that all topics in the interview guide were addressed, the moderators actively tracked the progression of themes during the interviews and introduced follow-up questions when necessary to return to underexplored areas. Situations in which group dynamics were characterised by clear dominance or power imbalances were rare. Nevertheless, the moderators continuously monitored interaction patterns, and on the few occasions when uneven participation was sensed, the discussion was gently redirected by inviting alternative perspectives (e.g., “How do others in the group view this?”) or by explicitly opening the floor to less vocal participants. This was done to support balanced participation while maintaining the natural flow of group interaction. Overall, the moderation strategy aimed to create an open discussion climate, ensure coverage of all core themes, and support the expression of perspectives within the group.

Transcription was conducted directly during the focus group interviews, ensuring that the transcripts used were both content-accurate and complete, which means that they are suitable as material for qualitative content analysis. However, in some instances, the exact wording has been slightly simplified and/or shortened due to a high speech rate. These transcripts form the basis for the analysis.

### Qualitative content analysis

For the purpose of the present study, to focus on how the participants make sense of the newly introduced ABW as reflected in working conditions, we conducted a qualitative content analysis based on Graneheim and Lundman (2003) to describe variations in the collected material (cf. Lindgren et al., 2020). The analysis involved two steps where the second step forms the basis for the study’s main findings on how the situation is made sense of by the participants. In the first step we analyse the manifest content to identify how working conditions were described, and developing categories and sub-categories related to the ABW setting. The second step, which is in focus here, concentrated on the latent content of the material collected, guided by the theoretical framework on sense-making (Weick, 1995). In this step, themes were analytically developed through the interpretative process in which recurring patterns of meaning across interviews were abstracted into higher-level concepts. These themes thus created represent shared views on how participants made sense of the new work environment. From this analysis process, categories and sub-categories describe the manifest content of the material, while themes, developed in the subsequent analytical step, capture latent patterns of meaning that cut across these descriptive levels and represent shared sense-making.

The analytical process began with a detailed examination of transcribed interviews by two of the researchers (first and last author) to establish a comprehensive understanding. Key excerpts, particularly those highlighting the transition from cellular offices to ABW, were identified and thoroughly analysed. Sections unrelated to the ABW setting were excluded. Following Graneheim and Lundman (2003), the research team iteratively evaluated transcripts to ensure each theme reflected discernible patterns. This process resulted in the agreed categories, sub-categories, and themes clarifying working conditions and sense-making in the ABW setting, presented in the results section.

### Research ethics

Prior to data collection, the study was reviewed and approved by the Swedish Ethical Review Authority (Etikprövningsmyndigheten), an independent authority not affiliated with any of the authors, in accordance with the Swedish Ethical Review Act (2003:460). Approval was granted under decision reference number 2023-06718-01. The study was conducted in compliance with national legislation, institutional requirements, and the principles of the Declaration of Helsinki.

All data collection and processing in the project followed the Swedish Research Council’s guidelines on research ethics (Swedish Research Council,^2017^), including information, consent, confidentiality, and data usage.

Before each focus group interview, participants received written and oral information about the study and the conditions for participation, with the opportunity to ask questions. Researchers confirmed that all participants had read and understood the information, emphasising the voluntary nature of participation. Informed consent was obtained through signed consent forms.

## Results

The first step of the analysis resulted in two categories describing the empirical findings on how the various staff groups experienced the **conditions of work** in newly introduced ABW in a municipal setting, highlighting both strengths and challenges related to the prerequisites for health and wellbeing at work, followed by subsequent sub-categories. The first part of the results gives a background to what it means to work in an ABW. Further, five themes were created from the second part of the analysis, uncovering how the interviewees made sense of the transition. These themes reflect five **dimensions of meaning-making**. These results constitute the study’s main findings where the themes describe the common interpretations of how the ABW is made sense of, found in the material running through the categories and bringing meaning to the phenomenon under study (cf. Lindgren et al., 2020). The results on conditions of work and dimensions of meaning-making, respectively, are presented in the following sections.

### Conditions of work

From the first step of the qualitative content analysis (cf. Graneheim & Lundman, 2003), categories relating to working conditions were created. From this manifest part of the analysis, it was found that the working conditions associated with the new ABW were defined and described in relation to strengths and challenges, respectively, which are also presented as the main categories of this part of the analysis, followed by sub-categories describing the aspects of working conditions found in the material. These results provide with context that enables the second step of the analysis to discern which work environment the participants make sense of. The results of the first step of the analysis are summarised below.

In term of perceived *strengths*, a central sub-category concerns how the ABW setting was constructed as enabling (1) increased interaction. The material conveys accounts of how the open layout appeared to facilitate encounters between colleagues who had previously remained distant. Informal communication practices were described as emerging more naturally, with the physical environment perceived as lowering barriers to spontaneous meetings. These interactions were often linked to narratives of enhanced collaboration and a livelier atmosphere. Another sub-category is (2) the experience of simplicity. The ABW space was portrayed as inviting and easy to navigate, with digital tools—such as systems for locating colleagues or booking workspaces—constructed as functional and user-friendly. Taken together, these accounts framed the ABW environment as contributing to (3) a broader sense of modernity, what constitutes the last sub-category within the category of strengths. In contrast to earlier depictions of a more traditional public sector workspace, the new setting was positioned as contemporary and stimulating, both physically and socially. Among the *challenges* identified in the material, a key theme in the material concerns how (1) the use of different zones within the ABW setting was often constructed as problematic, what is the first sub-category found in the material. The intended fluidity between zones rarely materialised, with the ‘quiet zone’ largely depicted as underused. The accounts instead describe a pattern of staying in the same place, which was associated with feelings of disconnection from the ABW concept. Another recurrent theme relates to time management, where (2) an increased focus on meetings was narrated as a challenge. Contrary to common expectations that ABW reduces the need for formal meetings, participants described a perceived rise in meeting frequency. Experiences of (3) weakened group cohesion also emerged as a sub-category from the analysis. The shift from team members being located together in designated rooms to being dispersed across the ABW space was portrayed as contributing to a sense of fragmentation. Further, (4) leadership and management were similarly constructed as more demanding, with accounts highlighting how the new spatial arrangements complicated oversight and everyday contact. In some narratives, concerns were raised regarding staff who might intentionally withdraw in this environment, particularly when sensitive issues such as substance abuse or other work-related difficulties were present. (5) Confidentiality was another prominent theme forming a sub-category in the material, especially among participants working within social services. While others referenced occasional insecurity, social services employees described tensions between their professional obligations and the realities of the open office layout. Even though smaller focus rooms were available, these were sometimes framed as insufficient in fully addressing the need for privacy. Finally, the material illustrates how the ABW environment was perceived as (6) posing ergonomic challenges, both physical and cognitive. Accounts emphasised disturbances from ambient noise and visual strain caused by design features such as dark privacy screens, contributing to a broader sense of discomfort in the workspace.

### Dimensions of meaning-making

In the second part of the qualitative content analysis (cf. Graneheim & Lundman, 2003), themes were created uncovering the latent meaning of the material analysed, and thus how the interviewees made sense of the new ABW setting what constitute the study’s main findings. Five themes representing dimensions of meaning-making were identified: Contrasting; Rationality; Collective process; Working from home; and Focusing on the ideal. These results are presented below, followed by quotations from the material illustrating the findings.

#### Contrasting

One way in which the personnel made sense of the transition to ABW was by contrasting the new situation with previous working conditions. This theme is demonstrated by the following quotations from one of the groups of employees (group IV):

*What I think they attracted us with... we were sitting in old premises—that it would be new, with new technology and digital solutions. That was where the appeal lay. A fresh start. (IP16)*

*It was necessary. When I arrived here. It's unbelievable, people were sitting like this! It was so old and worn out. (IP17)*

*I felt the same way when I started here. (IP18)*

*Super modern. (IP16)*

Generally, the material indicates a sense of optimism about working in an ABW. A sense of anticipation was expressed regarding the new approach to working, particularly with respect to the new premises. There was a notable sense of joy about finally transitioning to a fresh and modern environment, which stood in stark contrast to the experience of the previous workspace, as expressed in the following quotation from one of the managers (group II):

*There's something quite lovely about the backpack being the office. A bit modern. Bringing in younger people who have never even worked in a private office. Feels more natural. (IP7)*

As illuminated by this first theme, the transition from traditional cellular offices to ABW was framed by the interviewees as a shift marked by positive anticipation and stark contrast to their previous working conditions. Initial challenges related to physical infrastructure, such as outdated facilities and fragmented office locations, were replaced by enthusiasm for modernisation, technological advancements, and a unified workspace. In the material, a sense of renewal and improved collaboration was highlighted, with fresh, open spaces fostering both spontaneous interactions and a more efficient workflow. The move was perceived as a necessary step towards a more contemporary, flexible, and socially integrated workplace, enhancing both job satisfaction and organisational cohesion, which constitutes a central perspective on how the situation was made sense of by the participants.

#### Rationality

Secondly, the transition was made sense of in terms of rationality, where ABW was understood as the only possible way forward, as mentioned here by one of the employees (group III):

*Yes, it's like this. I think all new workplaces will be like this. You can't justify anything else, as it would be too expensive. With the pandemic, we invited the home as a workplace, and now employers count on that. (IP12)*

*And so do employees. (IP11*)

The analysis reveals that meaning and acceptance are constructed by relating to a perceived rationality. The new way of working is viewed as efficient and economically justifiable, and is understood as a necessity, as demonstrated in the following summary of a dialogue between employees (group III):

*All the arguments have been presented. We're not so naive as to not realise it's about money.*

*They've talked about optimising the use of office space and we've experienced discussions about collaboration. They emphasised the desire to adopt a modern way of working.*

ABW is perceived as the only option, with cellular offices not even considered a meaningful topic for discussion. According to participants, a key argument in the transition to ABW was that the municipality must keep pace with societal progress. Against this backdrop, ABW is perceived as a rational choice. Here we observe a meaning-making process tied to a widely accepted idea of the modern workplace. Participants express that this approach to work is standard for all major organisations and that there is no viable alternative to this change, as mentioned here (group I, leadership positions):

*No one ever wants to go back to individual offices. The idea of expanding in 15 years just to provide private offices for everyone—that will never happen. You have to work towards getting more out of the space you already have. And the environmental aspect, making use of what you have. (IP1)*

The analysis also revealed that the management initially argued for ABW by emphasising its benefits and aesthetic appeal. However, this approach was met with resistance from employees. When the management instead clearly communicated that economic incentives were central, and framed the transition as a matter of responsibly managing taxpayers' money, the shift to ABW was more readily accepted within the organisation. This perspective can be seen in the following dialogue from one of the focus group interviews (group I, leadership positions):

*When you work in an organisation funded by taxpayers, you don’t want to pay for unnecessary rent. (IP2)*

*When we spoke with employees... We talked a bit too much about how nice it would be. That backfired a little. We pulled back and instead focused on costs. It’s not reasonable for us to have premises that we don’t fully utilise. Now we’re reaping the benefits now that we’re settled in. At first, people didn’t see the gains in collaboration. Everyone had different ways of working. But that has come back since then. This is taxpayers’ money. But today, you can see how nice it has turned out. People grow into the new environment. (IP4)*

Similarly, rationality was also seen as a foundational principle in instances where the transition to ABW faced resistance. The entire organisation worked over an extended period towards adopting this approach, and in cases where concerns and criticism arose—such as within the area of social services regarding the conditions for work under the new system—these issues were ultimately understood through the lens of rationality. The analysis reveals that the focus was not on identifying alternative solutions but rather on making the ABW model function in an acceptable manner within its established framework. Hence, even though it was evident from the analysis that the situation was made sense of in relation to rationality, resistance was also found in the material where the interviewees expressed that they felt forced to accept the situation, but that they believed ABW was unworkable for their part of the organisation.

#### Collective process

Further, a central basis for the understanding of the situation was the collective process that had unfolded over a long period of time. This perspective can be found in the following quotation (group IV, staff):

*One good thing is that it’s taken a very long time. The long lead-up has been helpful. But not everything is perfect. However, many of the problems they have at government agencies—we don’t have those. That could make a difference. (IP16)*

That is, the analysis highlights the importance of the prolonged notice period regarding the transition to ABW in the process of meaning-making. The municipality’s shift to ABW had been anticipated for several years, with information provided continuously through channels such as the intranet and meetings within individual workgroups. This ongoing dialogue is mentioned in the following quotation from one of the managers (group II):

*It has been okay to repeatedly raise concerns. But when we got to the investigations, when involving employees—what do we do at work, what do I need for my tasks—we said, ‘We’re going to do it this way. But I promise to do everything I can to ensure we can work safely.’ The previous premises were functional, but we weren’t going to stay there. Now we’re here, and I feel secure. When we transitioned to functional spaces, I felt secure, and that sense of security extends to the employees. It’s been an incredible learning process. Showing employees that they can influence things, that we listen, and that we solve issues together. (IP9)*

However, the material reveals variation in how effectively this information was perceived and the extent to which managers close to operations were seen as providing information to their employees. Although the material includes somewhat various perspectives, the most dominant view is that the process was well-functioning.

At the same time, there were instances in the material where the process was questioned to some extent, especially in the social services sector, as expressed here (group II, managers):

*We work with 99% confidentiality. How is that possible with people going behind your back? All the phone calls. But the first thing we were told was that it could be solved (in the usual premises). But eventually, it was decided that we would get a functional space. We’ve had to work a lot with the staff group, there’s been so much anxiety. Many small details have caused concern. We had a long time to prepare. But it’s turned out better than expected anyway. (IP9)*

*It was a decision made by the top politicians [to introduce ABW]. We had no say in the matter. We needed to move here. (IP24)*

To sum up, this theme illuminates how the transition to an ABW environment was made sense of in relation to the lengthy and inclusive process that aimed to build trust and alleviate employee concerns. Extensive mapping and continuous communication over several years allowed employees to voice their fears and influence the planning of new functional workspaces. While the transition was described in the material as well-functioning and empowering, particularly in terms of fostering a sense of security and learning, some sectors—especially social services—faced greater challenges. Concerns about confidentiality and open workspaces required significant effort to address. Despite initial scepticism, the prolonged preparation period ultimately contributed to a smoother transition than anticipated, though some employees still felt the process was externally imposed.

#### Working from home

The analysis shows that the possibility of working from home was central for how the transition to ABW was made sense of in the focus group interviews. It is evident that the understanding of ABW is not confined to this work model within the municipality’s premises but is made comprehensible by being interpreted in relation to favourable conditions for many employees to combine work at the municipality’s offices with work from home, as discussed in the following dialogue among a group of staff (group IV):

*I think if we hadn’t been able to work from home... Home is my quiet zone. Personal matters, personal identification numbers. I don’t need to think about it. It would have been problematic if I had to be here all the time. At home, I can handle the sensitive parts, I can sit and talk. But if everyone is expected to do that on-site, then they would need to expand. (IP17)*

*Yes. Being at work gives me more than being at home, because of the colleagues. I use my wellness time at home. (IP15)*

*I work from home because it’s my quiet zone. I haven’t used a quiet zone at work, because I do that at home. But also, with age, you get a bit mentally tired. It works so well because I can rest my brain at home. I might still be at the office five days a week. But I notice that having people around is important. (IP16)*

In the material, ABW is thus understood as part of a broader solution that includes remote work. This also involves the home being specifically used as a ‘quiet zone’ or as an option for employees seeking a calm and peaceful work environment, in contrast to the dynamic and lively atmosphere within the ABW setting, as expressed here (group III, staff):

*The combination is what I like—being able to work from home and also get some mental recovery. It would be really boring to work from home all the time. (IP11)*

*Yes, it was horrible. You get energy in the office but need the retreat at home. (IP13)*

However, not all interviewees stated that they had the possibility to work from home, for example those working with confidential issues as part of social services and administrative personnel. This perspective on working from home is exemplified in the following quotation from one of the managers (group II):

*In our organisation, there is a clear directive that we should be at the town hall, we should be visible, we should be on-site. But that directly contradicts productivity and the employees' work environment. If you’re supposed to deliver something, you have to be at home. (IP7)*

Hence, those working from the ABW all or most of the time seem to hold more negative views on the ABW than others. The analysis also highlights perceived inequities, where it remains unclear what determines which employees are allowed to work from home and to what extent remote work is approved. In some cases, there is a sense that the conditions granted to a particular workgroup depend on the personal preferences of the immediate supervisor, as expressed by some of the staff in the quoted discussion here (group V, staff in social services):

*It varies depending on which team you work in. There’s probably a policy. (IP25)*

*We in [specific department] can’t work from home at all. (IP24)*

*It’s clearly stated that we must be available. If you work from home, you can’t take confidential calls, so you can’t be available for clients. This must be confirmed with the manager. (IP23)*

*We have a [maximum number of days allowed to work from home]. (IP20)*

*Different rules depending on which manager you have—but everyone has the same sector manager. I can have confidential calls at home, but you can’t. (IP25)*

*It was like we worked during the pandemic. Back then, everyone worked from home. (IP23)*

*Their [managers’] own opinions are what drives things. (IP25)*

*When we were moving here, with fewer spaces—many were supposed to work from home. It’s so strange that it’s different. (IP21)*

*It can’t be different under the same sector manager! (IP25)*

As highlighted by this theme, the integration of work from home emerged as a central aspect of how employees made sense of the transition to ABW. The analysis finds that working from home provided a crucial ‘quiet zone’ that complemented the dynamic and social environment of the office, allowing for mental recovery and tasks requiring focus and privacy. This hybrid model was seen as enhancing both productivity and well-being. However, significant disparities were highlighted, particularly among employees in social services and administrative roles, where working from home was often restricted due to confidentiality concerns and organisational policies. Such inequities, perceived as being influenced by managerial discretion rather than consistent policy, led to frustration and feelings of unfairness. The lack of adequate private spaces in the office further compounded these challenges, raising questions about the inclusivity and adaptability of the ABW model.

#### Focusing on the ideal

Lastly, a focus on the ideal of the ABW, rather than the reality, was found to be part of how interviewees made sense of the transition. As expressed in the following quotation, the zones are described as functioning in mind (group IV, staff):

*Does the zone system work? (Moderator)*

*The idea works, but it’s us humans who don’t follow the rules. (IP16)*

The analysis clarifies that the frame of reference used to interpret and understand the current situation with the ABW incorporates an understanding of ABW in terms of an ideal type. This perspective is revealed in the following quotation, where the interviewee emphasises that ‘everyone says’ that things are a certain way, but in practice, there is something else going on (group V, staff in social services):

*Everyone says it’s activity-based, but it’s mixed. They’ve combined different types of office landscapes and reduced the number of spaces. We don’t have enough spaces. (IP24)*

Meaning is thus constructed in relation to the ideal vision of ABW—how it is intended to function and how it has been described—rather than in relation to the actual conditions experienced in the current working environment. Further examples of this perspective can be found in the following excerpt, where one of the leaders of the steering committee describes challenges when it comes to sticking to the determined way of working (group I, leadership positions):

*The challenge is how to relate to the working method we’ve developed. What do the zones mean? How do we relate to the environments? We see when people walk around that they find their favourite spots and settle in the focus rooms, closing the door and staying there for hours. A great responsibility for managers. (IP4)*

In the following dialogue (group IV, staff), there is a discussion about the implementation of the ABW where the interviewees reflect on the difficulty to work as it is intended, and say that the concept is (already) in a need of rethink.

*Then the whole idea falls apart (if you don’t do it as intended) [---]. (IP17)*

*Yes, it’s time for a rethink of the rules for the zones. Now you can see that informal decisions have been made, and it’s okay to talk and joke around. The same goes for the focus rooms. A rethink is needed. And that’s probably what needs to happen. (IP16)*

To sum up, this last theme shows that the transition to ABW is interpreted through an idealised vision rather than the actual experience. In this way, the analysis reveals that the idealised image of what ABW could be is used as a tool to make sense of the situation. While participants express an appreciation for the concept itself, they also articulate significant challenges with its practical implementation. While some zones, such as the quiet zone, function effectively, others, like the middle zone, are used in ways that deviate from their intended purpose. This has led to the emergence of informal rules and behaviours, with some employees ‘claiming’ rooms or spaces, causing frustration and limiting availability. Also, the management emphasises the need to return to the original guidelines for zone usage. Although the ABW model promotes activity-based working and flexibility, many employees choose workspaces based on personal preferences rather than the requirements of the task. The analysis finds that for the model to function as intended, continued leadership and clearer rules are necessary to balance individual needs with organisational goals.

## Discussion

In the present study, we have analysed the experiences of leaders and employees from various departments in a municipal setting regarding working conditions related to a newly introduced ABW. The results of the qualitative content analysis (cf. Graneheim & Lundman, 2003) describe how the participants make sense of the new ABW setting as reflected in working conditions.

In the first step of the analysis, we created categories (strengths and challenges, respectively), followed by sub-categories that describe what was found in the material regarding how the new working conditions were discussed by the interviewees what mostly align with results from other studies (cf. Hua et al., 2020; Laing, 2017; Pettersson-Strömbäck et al., 2018; Seddigh et al., 2015; Truex et al., 2019). However, in the present study, these results are to be understood as a background for the second step, what constitute the focus of the study. Hence, the second step of the qualitative content analysis focused on how the interviewees made sense of the situation (cf. Weick, 1995), that is the latent meaning of the material, specifically regarding the process of clarifying and understanding the new ABW setting.

The results showed that, through interaction, the participants developed a common understanding of the situation that arose (cf. Maitlis & Christianson, 2014; Weick, 1995), based on five distinct themes representing dimensions of sense-making: Contrasting, Rationality, Collective process, Working from home, and Focusing on the ideal. These dimensions were present across all focus group interviews and together constitute an analytically derived framework through which the ABW was collectively made sense of.

As highlighted within the theoretical framework, people may differ in how they make sense of a situation, which can be influenced by previous experiences and expectations that vary between groups within an organisation (cf. Kihlberg, 2022). Therefore, it is important to consider whether such variations exist in the material analysed here. It is often argued that such variations appear between individuals from groups with different status within the organisation; for instance, between managers and employees (cf. Brown et al., 2008). However, the analysis reveals that the variation here occurs more between sectors. That is, both managers and employees generally share the same framework for understanding the working conditions related to the new ABW setting, but those in leadership positions within the administrative area of social services differ in their perspectives.

As mentioned in the results section, the analysis highlights how the interviewees from social services felt compelled to accept the new working environment (Rationality; Collective process) and diverged from other employees in terms of sense-making, particularly in relation to the theme ‘Working from home’. This group was not permitted to work from home due to the nature of their duties, which involve confidential matters. This exclusion created a sense of inequality and reinforced a divide between social services and other sectors, as well as between the rhetoric of flexibility and employees' lived experiences. Furthermore, the open-plan design and non-territorial workspace—hallmarks of ABW—posed additional challenges for these employees, who struggled to find spaces appropriate for confidential discussions or emotionally demanding client work. As a result, the sensemaking process among this group was marked by a tension between rational acceptance of organisational change (Rationality) and a perceived lack of agency or participation in shaping the new working conditions (Collective process). These factors contributed to a more sceptical and at times critical stance towards ABW, compared to other sectors where employees experienced greater alignment between ABW principles and their day-to-day work. Thus, the analysis sheds light on how the sense-making process varies not only due to power relations and hierarchical positions within the organisation (cf. Brown et al., 2008; Kihlberg, 2022), but also due to area of responsibility.

The present study contributes further nuances to our understanding of variations in how organisational change—specifically, the newly introduced ABW in a municipality—is made sense of which is central to understanding the conditions for a healthy and sustainable working life. We have demonstrated that it is not only hierarchical position that matters (cf. Kihlberg, 2022) when it comes to sense-making, but also the characteristics of various working conditions across different areas of operation within the organisation. These factors also influence how organisational change is understood.

From this, the present study contributes to theory development by deepening the understanding of sensemaking (cf. Weick, 1995) in the context of workplace transformation, particularly regarding the introduction of activity-based working (ABW) in a municipal organisation. While prior research has frequently focused on the structural and material conditions of ABW (e.g., Hua et al., 2020; Pettersson-Strömbäck et al., 2018), fewer studies have explored how employees and managers cognitively and socially navigate the uncertainty and ambiguity that such transitions often entail.

We argue that the use of sensemaking theory is particularly relevant in contexts characterised by change and uncertainty (Maitlis & Christianson, 2014; Weick, 1995), such as the introduction of ABW, where established routines and spatial norms are challenged. In such situations, individuals and groups need to retrospectively and collectively construct meaning to reduce ambiguity and to act appropriately within the new system. ABW introduces both physical and cultural changes in the organisation that require not only adaptation to new office layouts and policies but also a redefinition of work identity, autonomy, and collaboration patterns (cf. Laing, 2017; Seddigh et al., 2015). In the present study, sensemaking theory enabled an exploration of how employees and leaders actively negotiated and constructed shared frameworks of understanding related to the newly introduced ABW. The themes identified demonstrate that such processes are central for explaining how individuals make the unfamiliar familiar, and how they legitimise or resist organisational change.

Importantly, the findings expand on existing theory by illustrating how sensemaking is not only shaped by formal power hierarchies (cf. Brown et al., 2008) but also by contextual factors such as the nature of work and operational area. This highlights the need to consider sector-specific constraints, such as confidentiality requirements in social services, which limit flexibility and affect how change is interpreted and enacted. Thus, the study advances theoretical understanding by showing that sensemaking in ABW transitions involves both organisational structures and area-specific working conditions influences. This nuanced perspective calls for a more context-sensitive application of sensemaking theory in future research on organisational change.

### Conclusion and implications

The conclusion of this study is that employees and managers within a municipal setting make sense of the newly introduced ABW in ways that are influenced by sector-specific conditions. By applying sensemaking theory, this study highlights the importance of considering not only hierarchical power but also the nature of work and sector-specific constraints when implementing organisational change. The findings call for more context-sensitive approaches to ABW, recognising that the benefits may not be equally applicable across different sectors.

For practitioners, the study indicates that ABW implementation within the public sector should be adapted to the specific requirements of different organisational contexts, particularly in social services. This involves ensuring access to suitable spaces for confidential tasks and considering flexible working arrangements that correspond to the nature of the work, in ways that promote employee wellbeing and contribute to a sustainable working life. Moreover, active involvement of employees in the change process is essential, not only for minimising resistance but also for creating organisational conditions that support health and long-term resilience.

### Methods discussion

This study used focus group interviews to collect material for analysing sense-making in the new ABW setting. While focus groups capture sense-making as a social process, they have limitations. Sense-making also draws on individuals’ prior experiences, where personal reflections interact with everyday workplace discussions. Thus, the research is limited to the conversations framed by the study, excluding ongoing organisational dialogues.

Regarding quality and credibility, participants were recruited internally via the municipality’s staff. This ensured a relevant sample but introduced potential bias. Employees might have been influenced by organisational roles or a desire to align with norms, possibly limiting the range of perspectives. They may also have hesitated to express criticism. Still, internal recruitment made the findings highly relevant to the organisational context, enhancing the study's practical value. To strengthen credibility, the study compared data across municipal sectors and included both managerial and employee perspectives. The rich qualitative data further supports the trustworthiness of the results by offering deep insight into participants' experiences and sense-making.

Despite limitations, the findings provide valuable insights into how sense-making differs across municipal operations and inform the implementation of ABW in this context.

## Data Availability

The data supporting the findings of this study are of a sensitive nature and contain information that could compromise the privacy of research participants. The participants did not provide consent for their data to be shared publicly. Therefore, the data are not openly available, but may be made available from the corresponding author upon reasonable request, subject to approval by the Swedish Ethical Review Authority (Etikprövningsmyndigheten) and in compliance with ethical and legal requirements.

## References

[cit0001] Babapour, M. (2019). From fading novelty effects to emergent appreciation of activity-based flexible offices: Comparing the individual, organisational and spatial adaptations in two case organisations. *Applied Ergonomics*, *81*, 102877. 10.1016/j.apergo.2019.102877 31422254

[cit0002] Bäcklander, G., Fältén, R., Bodin Danielsson, C., Toivanen, S., & Richter, A. (2021). Development and validation of a multi-dimensional measure of activity-based working behaviors. *Frontiers in Psychology*, *12*, 655881. 10.3389/fpsyg.2021.655881 34744852 PMC8563580

[cit0003] Balogun, J. (2006). Managing change. Steering a course between intended strategies and unanticipated outcomes. *Long Range Planning*, *39*(1), 29–49. 10.1016/j.lrp.2005.02.010

[cit0004] Balogun, J., & Johnson, G. (2004). Organizational restructuring and middle manager sensemaking. *Academy of Management Journal*, *47*(4), 523–549. 10.2307/20159600

[cit0005] Balogun, J., & Johnson, G. (2005). From intended strategies to unintended outcomes. The impact of change recipient sensemaking. *Organization Studies*, *26*(11), 1573–1601. 10.1177/0170840605054624

[cit0006] de Been, I., Beijer, M., & Boonstra, A. (2015). Investigating the micro-foundations of collaboration in an activity-based work environment. *Facilities*, *33*(11/12), 636–651.

[cit0007] Berger, P. L., & Luckmann, T. (1967). The social construction of reality. *A treatise in the sociology of knowledge*. Garden City.

[cit0008] Bodin Danielsson, C., & Bodin, L. (2008). Office type in relation to health, well-being, and job satisfaction among employees. *Environment and Behavior*, *40*(5), 636–668.

[cit0009] Bodin Danielsson, C., Bodin, L., Wulff, C., & Theorell, T. (2015). The relation between office type and workplace conflict: A gender and noise perspective. *Journal of Environmental Psychology*, *42*, 161–171. 10.1016/j.jenvp.2015.04.004

[cit0010] Bosch-Sijtsema, P. M., Ruohomäki, V., & Vartiainen, M. (2014). Activity-based office concepts: A review of the literature. *Facilities*, *32*(3/4), 156–171.

[cit0011] Bouvier, C., & Eriksson, J. (2014). Framtidens arbetsplats - riktlinjer för en implementering av aktivitetsbaserade arbetsplatser. Kungliga Tekniska Högskolan.

[cit0012] Brown, A. D., Stacey, P., & Nandhakumar, J. (2008). Making sense of sensemaking narratives. *Human Relations*, *61*(8), 1035–1062. 10.1177/0018726708094858

[cit0013] Brown, A. D., Colville, I., & Pye, A. (2015). Making sense of sensemaking in organization studies. *Organization Studies*, *36*(2), 265–277. 10.1177/0170840614559259

[cit0014] Brunia, S., & De Been, I. (2016). Accommodating new ways of working: Lessons from best practices and worst cases. *Journal of Corporate Real Estate*, *18*(1), 30–47. 10.1108/JCRE-10-2015-0028

[cit0015] Candido, C., Thomas, L., Haddad, S., Zhang, F., Mackey, M. G., & Ye, W. (2019). Designing activity-based workspaces: Satisfaction, productivity and physical activity. *Building Research and Information*, *47*(4), 1–15. 10.1080/09613218.2018.1476372

[cit0016] Colenberg, S., Jylhä, T., & Arkesteijn, M. (2020). The relationship between interior office space and employee health and well-being – a literature review. *Building Research and Information*, *49*(3), 352–366. 10.1080/09613218.2019.1710098

[cit0017] Eaton, N. (2019). Organizational change management: A critical review. *Journal of Organizational Change Management*, *32*(2), 203–222.

[cit0018] Gephart, R. P. (1993). The textual approach. Risk and blame in disaster sensemaking. *Academy of Management Journal*, *36*(6), 1465–1514. 10.2307/256819

[cit0019] Graneheim, U. H., & Lundman, B. (2003). Qualitative content analysis in nursing research. Concepts, procedures and measures to achieve trustworthiness. *Nurse Education Today*, *24*, 105–112. 10.1016/j.nedt.2003.10.001 14769454

[cit0020] Hardy, C., Lawrence, T. B., & Grant, D. (2005). Discourse and collaboration. The role of conversations and collective identity. *Academy of Management Review*, *30*(1), 58–77. 10.2307/20159095

[cit0021] Hedge, A. (2018). Workspaces that enhance human performance. *Ergonomics in Design*, *26*(2), 4–14.

[cit0022] Heerwagen, J., & Kinzie, M. (2007). *Environmental Impact on Creativity: A Study of Workspace Design*. Center for the Built Environment, University of California.

[cit0023] Hua, Y., Shao, L., & Lai, L. W. C. (2020). The impact of activity-based workplace on creativity: A study of knowledge workers in China. *Journal of Corporate Real Estate*, *22*(1), 86–101.

[cit0024] Jung, H., Joo, B. K., & Hong, T. (2020). The impact of activity-based office design on organizational and personal outcomes. *Journal of Corporate Real Estate*, *22*(3), 190–209.

[cit0025] Kihlberg, R. (2022). *Influence in sensemaking during change. A study of the Swedish Police reform and subsequent change work* [doctoral thesis, Umeå University].

[cit0026] Laing, A. (2017). Open-plan office layout is not just about costs and productivity; it also affects morale and turnover. *Journal of Corporate Real Estate*, *19*(1), 37–46.

[cit0027] Lawrence, T. B., Malhotra, N., & Morris, T. (2012). Episodic and systemic power in the transformation of professional service firms. *Journal of Management Studies*, *49*(1), 102–143. 10.1111/j.1467-6486.2011.01031.x

[cit0028] Lindgren, B.-M., Lundman, B., & Graneheim, U. H. (2020). Abstraction and interpretation during the qualitative content analysis process. *International Journal of Nursing Studies*, *108*, 103632. 10.1016/j.ijnurstu.2020.103632 32505813

[cit0029] Lüscher, L. S., & Lewis, M. W. (2008). Organizational change and managerial sensemaking: Working through paradox. *Academy of Management Journal*, *51*(2), 221–240.

[cit0030] Maina, G. W., Influence, S. K., & Sigana, F. M. (2019). Activity-based workplace design and its influence on productivity in organisations. *International Journal of Academic Research in Business and Social Sciences*, *9*(9), 979–998.

[cit0031] Maitlis, S. (2005). The social processes of organizational sensemaking. *Academy of Management Journal*, *48*(1), 21–49. 10.2307/20159639

[cit0032] Maitlis, S., & Lawrence, T. B. (2007). Triggers and enablers of sensegiving in organizations. *Academy of Management Journal*, *50*(1), 57–84. 10.5465/amj.2007.24160971

[cit0033] Maitlis, S., & Sonenshein, S. (2010). Sensemaking in crisis and change. Inspiration and insights from weick (1988). *Journal of Management Studies*, *47*(3), 551–580. 10.1111/j.1467-6486.2010.00908.x

[cit0034] Maitlis, S., & Christianson, M. (2014). Sensemaking in organizations. Taking stock and moving forward. *Academy of Management Annals*, *8*(1), 57–125. 10.1080/19416520.2014.873177

[cit0035] Marzban, S., Candido, C., Mackey, M. G., Engelen, L., Zhang, F., & Tjondroegoror, D. W. (2023). A review of research in activity-based working over the last ten years: Lessons for the post-COVID workplace. *Journal of Facilities Management*, *21*(3), 313–333. 10.1108/JFM-08-2021-0081

[cit0036] Masoudinejad, S., & Veitch, J. A. (2023). The effects of activity-based workplaces on contributors to organizational productivity: A systematic review. *Journal of Environmental Psychology*, *86*, 101920. 10.1016/j.jenvp.2022.101920

[cit0037] Mumby, D. K. (2005). Theorizing resistance in organization studies. A dialectical approach. *Management Communication Quarterly*, *19*(1), 19–44. 10.1177/0893318905276558

[cit0038] Nieuwenhuijsen, E. R., & Topcu, A. (2019). The influence of organizational support and job autonomy on the relationship between activity-based workplace and employee job satisfaction. *International Journal of Human Resource Studies*, *9*(1), 1–22.

[cit0039] Pettersson-Strömbäck, A., Bodin Danielsson, C., Nordin, M., Öhrn, M., Harder, M., Olsson, T., & Wahlström, V. (2018). *Slutrapport från Aktikon-projektet i Örnsköldsviks kommun*. Umeå universitet, Instituitonen för Folkhälsa och klinisk medicin.

[cit0040] Potter, J., & Wetherell, M. (1987). *Discourse and social psychology. Beyond attitudes and behaviour*. Sage.

[cit0041] Richardson, L., & West, J. (2016). Spaces that enhance learning: Insights from science park tenants. In Hodgson, V. et al. (Eds.), *The design, experience and practice of networked learning* (pp. 103–122). Springer.

[cit0042] Sandberg, J., & Tsoukas, H. (2015). Making sense of the sensemaking perspective. Its constituents, limitations, and opportunities for further development. *Journal of Organizational Behavior*, *36*(S1), S6–S32. 10.1002/job.1937

[cit0043] Schildt, H., Mantere, S., & Cornelissen, J. (2019). Power in sensemaking processes. *Organization Studies*, *41*(2), 241–265. 10.1177/0170840619847718

[cit0044] Seddigh, A., Stenfors, C., Berntsson, E., Bååth, R., Sikström, S., & Westerlund, H. (2015). The association between office design and performance on demanding cognitive tasks. *Journal of Environmental Psychology*, *42*, 172–181. 10.1016/j.jenvp.2015.05.001

[cit0045] Smircich, L., & Morgan, G. (1982). Leadership. The management of meaning. *Journal of Applied Behavioral Science*, *18*(3), 257–273. 10.1177/002188638201800303 10260212

[cit0046] Stöllman, Å., Eriksson, T., & Vingård, E. (2015). *Flytten till Psykiatrins Hus – Arbetsmiljön i Öppna Arbetsplatser*. Uppsala universitet.

[cit0047] Swedish Research Council. (2017). *Good Research Practice*. Stockholm: Swedish Research Council.

[cit0048] Thomas, R., Sargent, L. D., & Hardy, C. (2011). Managing organizational change. Negotiating meaning and power-resistance relations. *Organization Science*, *22*(1), 22–41. 10.1287/orsc.1090.0520

[cit0049] Truex, D., Kruse, M., & Bodner, D. (2019). Serendipity, space, and innovation in a corporate R&D center. In Chen, J, et al. (Eds.), *The Routledge companion to innovation management* (pp. 343–360). Routledge.

[cit0050] Tsoukas, H. (2005). Afterword: Why language matters in the analysis of organizational change. *Journal of Organizational Change Management*, *18*(1), 96–104. 10.1108/09534810510579878

[cit0051] Wall, E. (2014). Sense-making of risk and role-taking emotions. How young Swedes construe road traffic risk. *Journal of Risk Research*, *17*(10), 1285–1299. 10.1080/13669877.2013.879487

[cit0052] Wall, E., & Olofsson, A. (2008). Young people making sense of risk. How meanings of risk are materialized within the social context of every-day life. *Young*, *16*(4), 431–448.

[cit0053] Weick, K. E. (1969). *The social psychology of organizing*. Addison-Wesley.

[cit0054] Weick, K. E. (1995). *Sensemaking in organizations*. Sage.

[cit0055] Weick, K. E., Sutcliffe, K. M., & Obstfeld, D. (2005). Organizing and the process of sensemaking. *Organization Science*, *16*(4), 409–421. 10.1287/orsc.1050.0133

